# Anæsthetisation for Operations on the Bladder and Urethra

**Published:** 1909-01-23

**Authors:** 


### Anæsthetics.

### ANÆSTHETISATION FOR OPERATIONS ON THE BLADDER AND URETHRA.

The greater part of the article on "Anæsthetisation for Operations within the Abdomen" (The Hospital, April 11, 1908) is applicable to the cases now under consideration. It is equally necessary to select the anæsthetic according to the age and physique of the patient, and the nature and probable duration of the operation: also to bear in mind the need for muscular relaxation, and the importance of avoiding cough and vomiting. As prolonged or severe operations are sometimes performed, and as patients needing operations in this region are apt to be in bad condition for general anæsthesia—being often worn out with pain and want of sleep, anæmic from loss of blood, or affected by alcoholism, sepsis, or renal inadequacy—and as a deep anæsthesia may be required to avoid reflex disturbances starting from the highly sensitive urinary tract, it is evident that the task of the administrator is frequently attended by much responsibility and anxiety. No operation should be started until the anæsthetist considers the patient "ready." Even the passage of a catheter may put a sensitive subject imperfectly anæsthetised at once into a dangerous condition.

*Litholopaxy.*—It is important that the anæsthesia should be such as to prevent straining or coughing, which is not only inconvenient to the operator but also dangerous to the patient. Too light anæsthesia has resulted in injury of the bladder by the instrument, and even in its rupture.

*Cystoscopy.*—In some cases there is so much intolerance of instrumentation or of distension of the bladder that deep general anæsthesia must be induced. This is especially so when tuberculous ulceration exists. Care must, of course, be taken that neither the anæsthetic nor the distension be pushed to the point of danger. It is very desirable to avoid coughing and straining, not only for the same reasons as in lithotrity, but also because hæmorrhage from the interior of the bladder may be set up and render a view impossible.

*Supra-pubic Cystotomy.*—Here, again, a quiet anæsthesia is imperative, particularly when the peritoneum is reflected low down and lies exposed in the upper part of the wound. *Vesical growths* are now usually dealt with in the Trendelenburg position, which has its advantages from the anæsthetist's point of view, especially in anæmic subjects.

*Supra-pubic Prostatectomy.*—This operation commonly affords a severe test of the carefulness, skill, and experience of the anæsthetist. On account of the degenerations of the circulatory and respiratory organs attending old age ether is usually quite unsuitable, and chloroform—or, in feeble subjects, A.C.E. or C.E.—should be given.

It is often found that the stage during which the anæsthetist has to pay the most attention is not, as might be supposed, that of enucleation, but that of distension of the bladder previous to cystotomy. A bladder which has for a long time been habitually over-distended and will hold without protest many ounces of residual urine sometimes greatly resents artificial distension on account of its comparative rapidity. Under the deep anæsthesia necessary for the purposes of the operator, the straining that would otherwise occur is represented by hesitating breathing with prolonged expiration, which in these old men is often accompanied by disturbance of the pulse: this may even go on to a stoppage of both pulse and respiration. If this condition is threatened the surgeon should be warned and asked to allow some reduction of the distension and open the bladder as quickly as possible; and if failure have occurred the bladder should be at once emptied in the quickest way practicable.

The process of enucleation usually acts as a powerful stimulus, respiration being deepened and the pulse becoming fuller. If, however, the patient be feeble or the enucleation prolonged (as may happen when the prostate is hard and adherent), the usual signs of depression come on sooner or later.

Much difficulty may occur from spasmodic contraction of the recti abdominis and levatores ani, which may be accompanied by that of the muscles of the lower jaw, tongue, and larynx, making it necessary to insert a gag and pull out the tongue. This may happen in spite of deep anæsthesia, just as it does in some operations on other sensitive parts. The contraction of the recti may prevent the surgeon reaching the prostate with his finger, and even if he can do so that of the levatores seriously hampers his movements. In such cases it is better for him to desist from his efforts whilst the anæsthesia is cautiously deepened, ether being added to the chloroform if this has not been done already.

After completion of the operation, constant watching is required, as unexpected collapse or a transient recurrence of rigidity occasionally takes place. If the bladder has been opened above the pubes in any operation anæsthesia should be maintained until the wound has been well secured against straining.

*Perineal Operations.*—When a patient is in the "lithotomy position" the anæsthetist must see that no pressure is put on the abdomen when the thighs are flexed. This is apt to occur in fat people, and when there are thick blankets over the abdomen it may seriously embarrass the action of the heart and lungs.

It is possible that in the future general anæsthesia will be replaced by spinal analgesia for many of these operations. Patients requiring prostatectomy are often bad subjects for a general anæsthetic under any circumstances, and the more so having regard to the need for a deep anæsthesia and to the special danger which has been described. Under spinal analgesia there has been, in the few cases so far carried out by the writer, complete muscular relaxation, which has added greatly to the ease and rapidity of the operation, and after-effects, including shock, have been conspicuously absent. Except, perhaps, for this operation, it seems advisable to limit its use at present to those cases in which there is some unusual risk in giving a general anæsthetic, and the patient should always be prepared for general anæsthesia in case the spinal method fails.

